# Safer school with near-UV technology: novel applications for environmental hygiene

**DOI:** 10.1007/s40201-023-00850-5

**Published:** 2023-01-26

**Authors:** G. Messina, R. Bosco, D. Amodeo, N. Nante, I. De Palma, C. Petri, G. Cevenini

**Affiliations:** 1 Department of Molecular and Developmental Medicine, University of Siena, Siena, Italy; 2 Post Graduate School of Public Health, Department of Molecular and Developmental Medicine, University of Siena, Siena, Italy; 3 Department of Medical Biotechnologies, University of Siena, Siena, Italy

**Keywords:** 405 nm, Near-UV, Environmental hygiene

## Abstract

Systems capable of disinfecting air and surfaces could reduce the risk of infectious diseases transmission. Aim: to evaluate the effectiveness of near-UV LED ceiling lamps, with a wavelength of 405 nm, in improving environmental hygiene. Between November and December 2020, we conducted an experimental study having a pre-post design in a kindergarten room in Siena where 4 ceiling lamps with 405 nm LED technology were installed. Twice per day, sampling was performed before (T0) and after treatment with near-UV (T1). We used between 8 and 12 pairs of contact plates to sample at various random spots each day. Air samplings were also performed. The plates were incubated at 22 and 36 °C. Significance was set at 95% (p < 0.05). The mean level of Colony Forming Unit (CFU) at T(0) was 249 (95% CI 193.1 – 305.0) at 36 °C and 535.2 (374.3 – 696.1) at 22 °C. The reduction was significant at T(1): by 65% at 36 °C and, 72% at 22 °C. Also, for air contamination: 95.3% (98.4—92.3). A dose threshold of about 5 J/cm^2^ was identified to have an 80% CFU abatement and remains nearly constant. The advantage of being able to use this technology in the presence of people is very important in the context of controlling environmental contamination.

**Graphical abstract:**

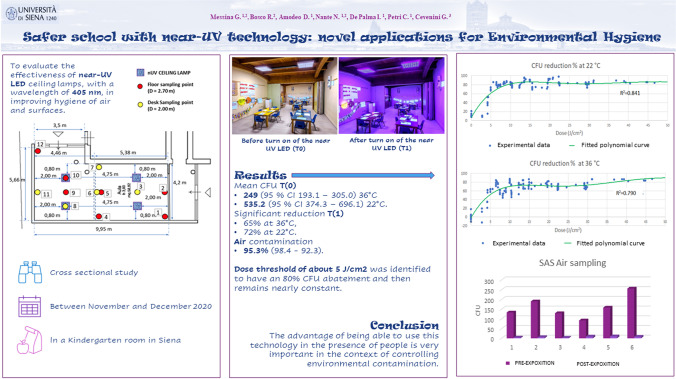

## Introduction

During the pandemic period, every closed place could be dangerous. The ECDC shared often communicates to invite people to stay at home, avoid crowded places and apply non-pharmaceutical intervention (NPI) [1]. Not only COVID-19 is a reason to evade assemblies or ignore hygiene practices. Several studies focused on influenza or other airborne infection and how these microorganisms have spread through social network [2], interpersonal relationships, and schools [3]. The many hours spent in a classroom with other people with different contacts networks increase the risk of an infection outbreak [3, 4]. Recently, we have seen politicians' difficulty in deciding whether closing schools is a good alternative. The control of viral transmission, especially in this case, is certainly fundamental, but at what price for students and workers? The ECDC says that the closure “should be used as last resort” [5], also because it alone is not sufficient to control the spread of the pandemic, as children often play an active role in the spread of this virus [6, 7]. But for future pandemics, the debate about closing schools might be 'easier' if these cultural and social places were safer through effective antimicrobial strategies. Traditional disinfection methods mainly consist of using harmful chemicals or heat [8]. Despite their easy use and effectiveness, these disinfection methods are not always eco-friendly. Especially in this particular period, it is important to research new disinfection technologies to contain the spread of viruses and other infectious agents. UV radiation has long proven to be effective in disinfection due to its germicidal properties [9]. Commonly used in the hospital setting, the pandemic has accelerated the need to introduce this disinfection strategy into home, workplace, and school environment, using UV LEDs and lamps. An innovative on-chip UV technology has also recently appeared [10]. The germicidal properties of UV-C (100-280 nm) are now well-known, but UV-C light cannot be used in the presence of people and in the long run tends to deteriorate various materials [11, 12].

Only recently have researchers focused on the inactivation of microorganisms by visible light, particularly in the near-UV (nUV) part of the spectrum, which is characterized by blue-violet colors in the wavelength range of 400 to 440 nm. Although not as bactericidal as ultraviolet light, visible shortwave light has several advantages, such as especially much greater biosafety for users and reduced photodegradation of materials [13, 14]. nUV is also used for medical treatment, such as infections and cancer [15, 16]. The main limitations of nUV technology are the low bactericidal effectiveness in shaded areas (when light is not direct) and the need of very long exposure times for the decontamination of UV-resistant bacterial strains and spores [17]. The germicidal effect of blue light occurs through photodynamic inactivation due to the photoexcitation of endogenous porphyrins, which act as photosensitizers. Porphyrin molecules, excited by the light, react with oxygen and cellular components to produce reactive oxygen species (ROS) that induce an accumulation of oxidative damage to the extent of cell death [18].

This study aims to evaluate the effectiveness of an nUV LED ceiling light in disinfecting the air and surfaces in a kindergarten at night when no people are present.

## Materials and methods

### Setting and study design

This experimental study, having a pre-post design, was conducted between November and December 2020 in 13 days in a room (about 47 m^2^) at “San Benedetto” kindergarten placed in Siena (Italy).

**Fig. 1:**
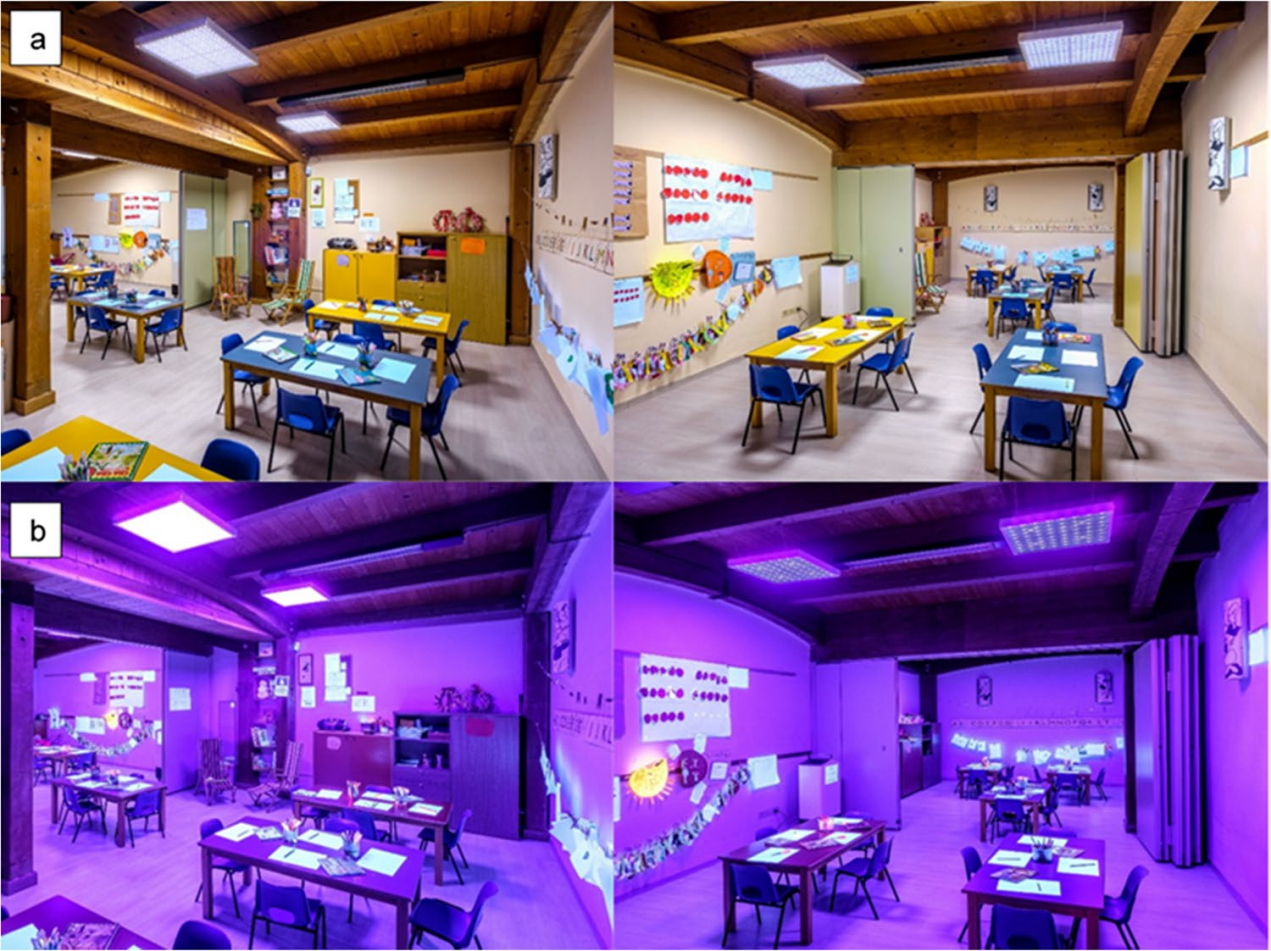
Kindergarten room with normal illumination (**a**) and nUV light (**b**)

Four ceiling lamps were installed in one room (Figs. 1a and b). Bacterial contamination sampling points were placed in the floor, 2.70 m from the lamps. Other detection points were placed above some desks about 70 cm high.

### Ceiling lamp

Each lamp consists of an array of 68 white LEDs (Nichia NVSW219FT) and 12 nUV at 405 nm (Luminus SST-10-UV) produced by Ugolini & C srl. The two types of LEDs can be used separately but also simultaneously to have both the effect of illumination (white LEDs) and disinfection (nUV LEDs). It was decided to turn on only the white LEDs during the daily activities and the nUV LEDs in the evening (during closing hours), after the routine disinfection operations, without the presence of operators. This alternation has been adopted as a principle of precaution since the devices were in prototype form. The nUV LEDs were controlled by a timer that automatically turned them on at 7:00 pm and turned off at 7:00 am the following morning.

### Stages of activities

**Fig. 2:**
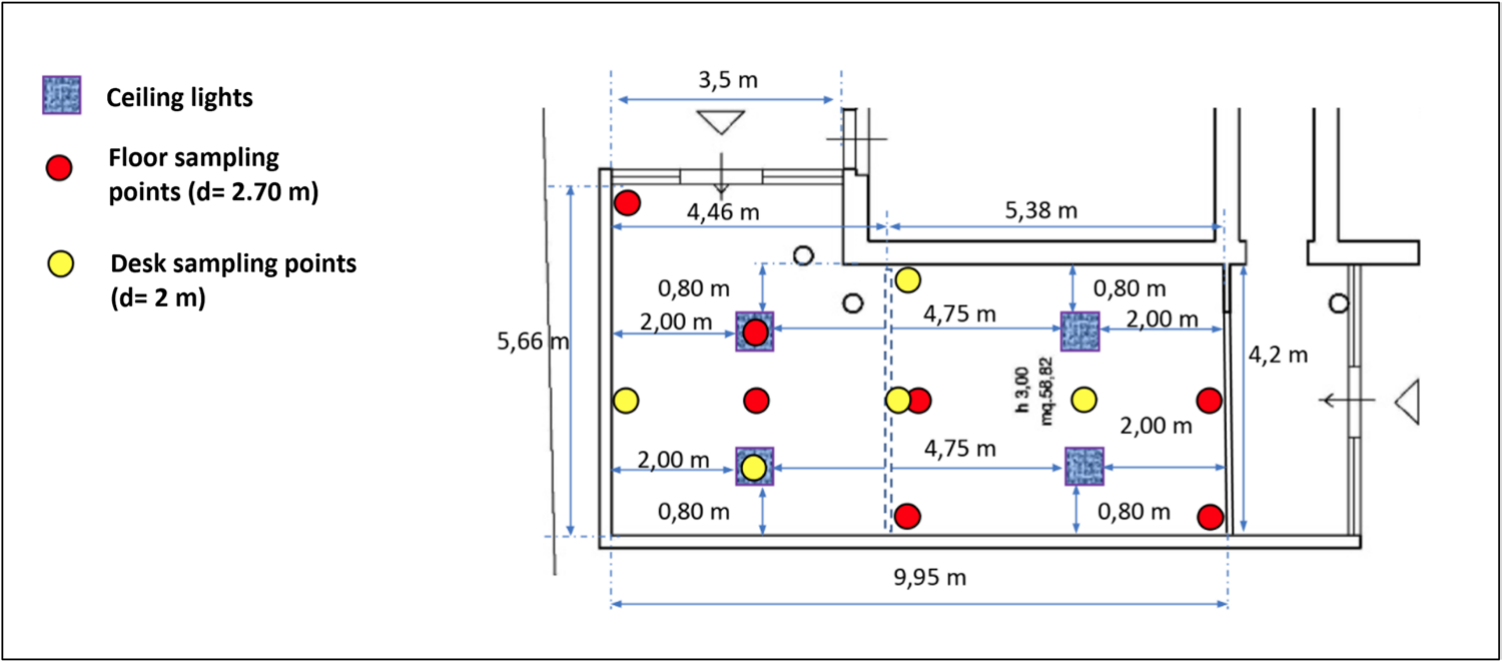
Schematic representation of the real space setting of kindergarten

In a preliminary stage, we selected 12 positions and measured the irradiance and the radiant flux, with a portable spectrophotometer (AvaSpec-ULS2048CL-EVO-USB3, Avantes, Apeldoorn, Netherlands), in every spot (Fig. 2). Surfaces were sampled by using contact plates (Rodac), filled with generic plate count agar medium (Oxoid, Basingstoke, United Kingdom). Two samples were carried out for each spot to incubate at 22 and 36 °C.

During the first seven days, sampling was carried out on the 12 selected spots. We subsequently reduced the number of spots from 12 to 8, using only the most representative ones and randomizing daily sampling in the different exposure areas. Different exposure times were selected: 8, 12, and 36 h. Microbial contamination was measured at each selected spot before system turn-on, at T(0), and after nUV treatment. As in the preliminary phase, two Rodac plates were used to sample each spot which were then incubated at two different temperatures (22 °C and 36 °C) to promote the growth of psychrophilic and mesophilic microorganisms. We also covered the area next to each spot with a white opaque plastic lid and performed post-exposure sampling to assess the real nUV germicidal effect and exclude confounding environmental factors. As a final control, to determine the environmental microbial population in the room, we performed 2 additional sampling days, maintaining the same spots unchanged but keeping the nUV lights off all night long. For 13 days, 2 m^3^ of air from the sealed room were aspirated at a speed of 120 L/min using two microbiological air samplers (Sas Microflow α Aquaria), which were set to automatically start before and after the nUV light exposure, to estimate the reduction of the environmental microbial contamination in the air. Then, the plates were incubated at 36 °C for 48 h. During nUV exposure, the room had closed doors and windows.

For microbiological reduction of surface contamination, descriptive statistics were performed including the stratification by doses (irradiance and time of exposition) into quartiles (25, 50, and 75 percentiles). Results were expressed as the percentage reduction, mean differences, and logarithmic reduction mean with a 95% confidence interval. A moving average filter with 10 points was developed to smooth Colony Forming Unit (CFU) reduction data as a function of the dose. The data were organized by associating the doses with the percentage reduction in bacterial load and sorting them by increasing doses. The controls (dose = 0), were used for statistical comparison with the nUV irradiated data. To test at what dose the differences between the irradiated data (sorted by increasing doses) and the non-irradiated controls were statistically significant at 95%, we repeatedly applied the Mann–Whitney test with moving windows of 10 data points for successive dose values. To account for statistical dependence between successive comparisons, we used Bonferroni correction. Two polynomial curves were fitted to experimental data at 36 and 22 °C. Mean and percentage pre-post CFU reduction and 95% CI were calculated on environmental air samplings. To test whether confounding factors could influence any difference in microbiological counts in air, before and after treatment, pre-post sampling was also performed with the ceiling lamps off. The Wilcoxon test was used to evaluate the statistical differences between T(0) and T(1) at the 95% significance level. Analysis was conducted using Matlab R2021a and Stata Ver. 16 software.

## Results

Table 1 shows the irradiances in the sampling points.

**Table 1:** Irradiance measured at selected exposure points

Point	Irradiance (µW/cm^2^)
1	172.0
2	148.6
3	674.8
4	231.1
5	240.6
6	359.7
7	312
8	682.9
9	519.1
10	515.4
11	284.3
12	96.4

A total of 520 Rodac plates were used for the entire length of the study: 130 matching points, 130 Rodac plates at T (0) and 130 Petri dishes at T (1), incubated at 36 °C and, similarly, 130 matching points at 22 °C. The mean level of contamination at T (0) was respectively 249 CFU (95% CI 193.1 – 305.0) at 36 °C and 535.2 CFU (374.3 – 696.1) at 22 °C; at T (1) we had a mean significant (p < 0.001) reduction both at 36 and 22 °C. At 36 °C we had a mean of 87.4 CFU (56.3 – 118.6), and at 22 °C 149.6 CFU (83.7 – 215.4). In percentage the reduction between T (0) and T (1), for the whole study period, was 65% at 36 °C and 72% at 22 °C.

The first, second, and third quartiles (25, 50, and 75 percentiles) had respectively, doses up to 6.93, 13.08, and 22.28 J/cm^2^. Table 2 summarises the microbiological results, stratified by dose range.

**Table 2:** Pre-post mean CFU comparison within quartiles

Dose range		Incubation temperature [°C]	Exposition	Mean [CFU]	Standard Error	95% Confidence Interval			CFU percentage reduction [%]	*P*-value [mean (diff) ˃ 0]
[quartiles]	[J/cm^2^]					lower bound	-	upper bound		
Q0 - Q1	2.78 - 6.93	22	Pre-Exp	1,135	312	492	-	1,778	63.6	0.0014
			Post-Exp	413	144	116	-	710		
		36	Pre-Exp	392	90	208	-	576	40.0	0.0024
			Post-Exp	235	66	100	-	371		
Q1 - Q2	6.93 - 13.08	22	Pre-Exp	481	115	248	-	714	75.9	0.0001
			Post-Exp	116	38	38	-	193		
		36	Pre-Exp	247	45	156	-	338	72.4	0.0001
			Post-Exp	68	16	35	-	101		
Q2 - Q3	13.08 - 22.28	22	Pre-Exp	473	687	22	-	721	83.6	0.0004
			Post-Exp	77	142	26	-	129		
		36	Pre-Exp	188	50	87	-	289	76.3	0.0010
			Post-Exp	45	13	18	-	71		
Q3 - Q4	22.29 - 88.50	22	Pre-Exp	212	49	113	-	310	72.9	0.0011
			Post-Exp	57	15	28	-	87		
		36	Pre-Exp	202	44	112	-	292	81.9	0.0001
			Post-Exp	38	10	17	-	59		

**Fig. 3:**
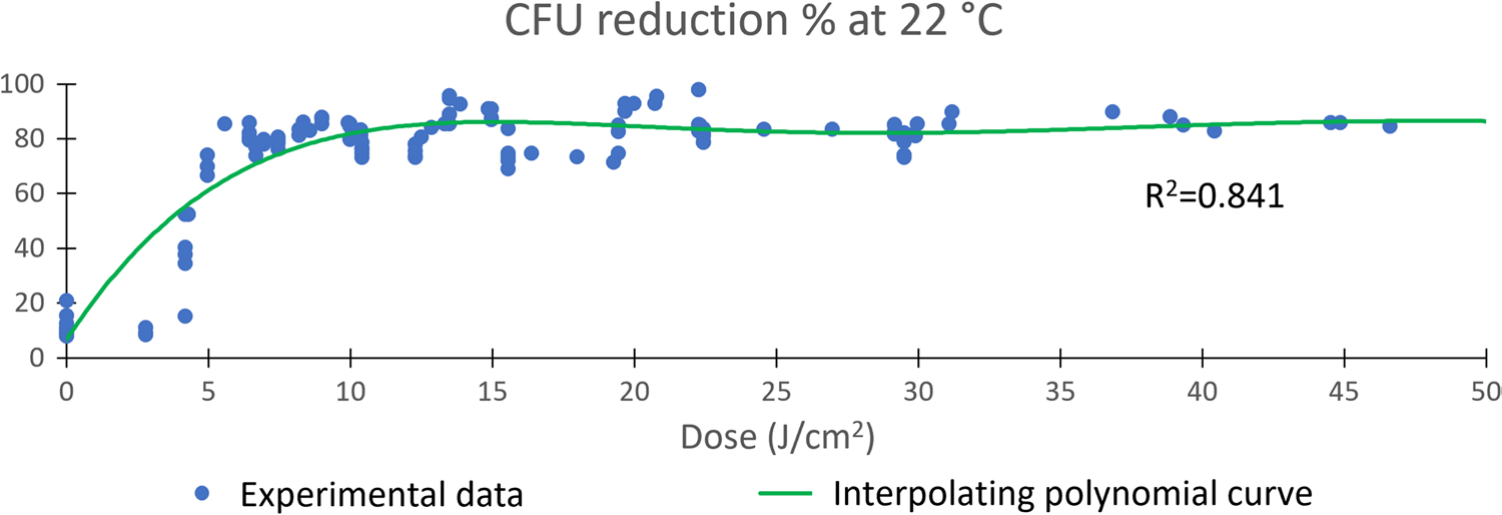
Dose-dependent CFU percentage reduction after nUV exposure of the samples incubated at 22 °C. R^2^ ranges from 0 to 1 and represents the fitting accuracy (0 = null; 1 = perfect) of the polynomial model (green line)

**Fig. 4:**
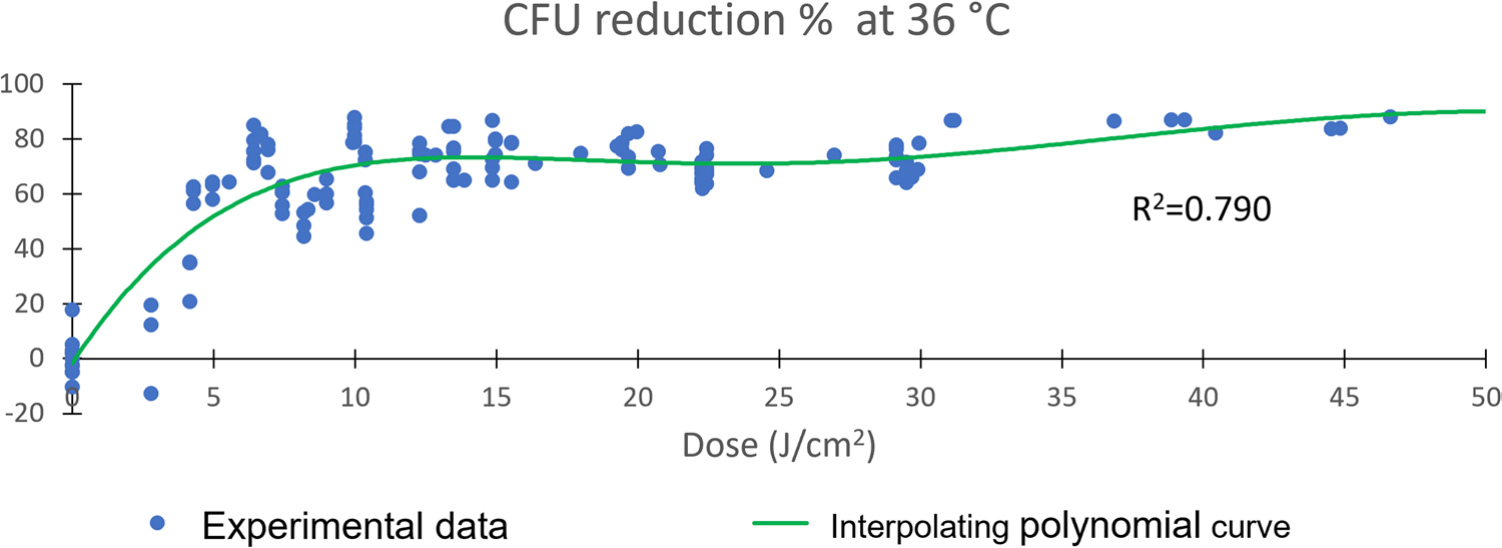
Dose-dependent CFU percentage reduction after nUV exposition of the samples incubated at 36 °C. R^2^ ranges from 0 to 1 and represents the fitting accuracy (0 = null; 1 = perfect) of the polynomial model (green line)

As far as nUV radiation doses (J/cm^2^) are concerned, a threshold was identified for both samples incubated at 22 and 36 C° of about 5 J/cm^2^, where a percentage of about 60% CFU reduction is statistically significant (Bonferroni correction to Mann–Whitney test, p < 0.05) and beyond which the system tends to a plateau value of between 80 and 90% CFU reduction (Figs. 3 and 4).

**Fig. 5:**
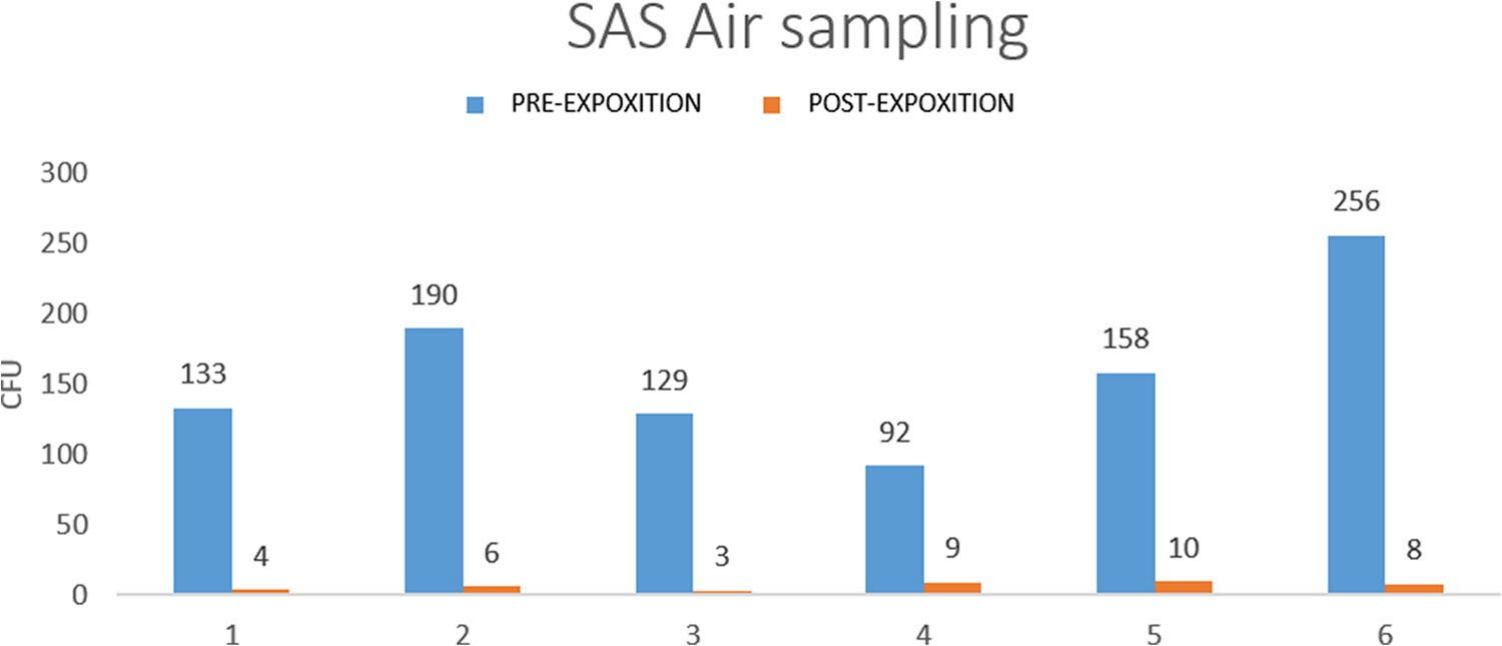
Air sampling pre/post nUV exposure

Figure 5 shows the air sampled CFUs, using the SAS and incubating at 36C°, pre-post use of the ceiling lamp. Mean CFU reduction on air samplings was 153 (93–213) corresponding to 95.8% (93.7%-96.8%). No significant differences (p > 0.05) were detected between T(0) and T(1) if the lamps were always off, confirming that the decrease of microbial contamination was influenced when the lights were on.

## Discussion

The use of systems that attenuate environmental contamination is becoming more widespread due to research demonstrating their role in preventing care-related infections [19, 20]. The scientific literature has focused primarily on disinfection of clinical and critical environments; however, the presence of individuals in confined spaces is an element that increases the risk of cross-contamination and potentially infection. Particular attention is emerging especially because of the recent SARS-CoV-2 pandemic. The setting and period of the investigation allowed for a study that would have been difficult to conduct in a non-pandemic scenario. The context of a kindergarten, where children are not wearing masks and have promiscuous behaviours, highlights, even more, the possibility of contamination; the disinfection system under consideration could help, assisted by routine cleaning, to increase hygiene levels.

The use of nUV light has proven optimal in consistently and substantially reducing airborne and surface contaminants. The results on surfaces allow us to observe a significant reduction in the environmental contamination in samples incubated both at 22 and 36 °C. Several studies confirm the efficacy of nUV: Maclean M. et al. had demonstrated that the gram-negative had required longer exposition than gram-positive [21].

The duration of exposure certainly improves environmental hygiene, a feature already noted by several studies [22, 23]. M. Maclean et al., in a study about the effects of high-intensity narrow-spectrum light (HINS-light) on staphylococcal contamination levels in hospital rooms, show that a period of extended light exposure leads to a significant reduction in bacterial load. Also, in the same study, the proportion of MRSA recovered isolates was significantly lower (62.5%) after exposure than before treatment (87.5%) [23]. In addition, decontamination of spores that may be present in the air requires longer exposure time at 405 nm and much higher doses of radiation than the previously mentioned microorganisms, as described by Maclean et al. in a study investigating the persistent problem of resistance of *Bacillus* and *Clostridium* endospores to disinfection treatments and their susceptibility to nUV light [17].

Another advantage of nUV is the absence of surfaces damage, as happens instead with UV-C light. This property is very important, especially for consecutive days of exposure, necessary for a better microbial load reduction [24, 25].

The nUV ceiling light proved to be an effective tool in reducing microbial growth in daily environments [19], when correctly designed to be organized in multiple source lighting systems, where some important parameters must be precisely defined depending on the spaces to be disinfected: i) the number of nUV sources and ceiling lights, ii) the exposure time, iii) the distance from the light source, iv) the light power and v) the obstacles in the environment.

As shown in Table 2, we found no direct proportionality between the dose supplied by the ceiling nUV light and the reduction of environmental contamination. Realspace experiments with nUV (405 nm) ceiling lamps on contaminated plates showed that microorganisms have resistance against this type of radiation non-proportional to the irradiation dose applied. A dose threshold of about 5 J/cm^2^ is identified before which the lamp is not effective and after which it rapidly reaches values of over 80% CFU abatement and then remains nearly constant (Fig. 3 and Fig. 4). Considering the environment in which the experiment took place, high doses of nUV radiation (J/cm^2^), over about 20 J/cm^2^, did not provide any further significant improvement in the level of disinfection, which already performs around 90% reduction in environmental microbial load. This is presumably due to non-specific microbial fauna in the environment. This makes nUV technology particularly suitable as a defensive barrier to the proliferation of microbial infections, which can also be used in the presence of people and pets [26–28]. The nUV, anyway, had demonstrated a good efficacy to reduce different types of microorganisms. In addition, different strains may respond differently to the same nUV dosage, depending on the environmental conditions that may influence different responses to irradiation and the conditions in which the same microorganism is treated [24]. In a comparative study, Murdoch et al. show how *Listeria monocytogenes* is more susceptible and rapidly inactivated than *Salmonella enterica* when treated on agar surfaces or in liquid suspension but less susceptible to 405 nm light when treated after drying on PVC and acrylic surfaces [24]. The inactivation data reported in this study can be compared with data obtained by Maclean et al. [21], which also reports that *L. monocytogenes* is less susceptible than most medically significant Gram-positive (i.e. *Staphylococcus, Streptococcus,* and *Clostridium* species).

It should be noted that the same radiant dose on commonly used surfaces, such as tables, chairs, tools, etc., which are much closer to the light source, can be achieved in considerably less time, especially if the triggering effect is considered.

Despite the aforementioned advantages of using this type of radiation, we have to remember that it does not substitute sanitation procedures of surfaces, which remain a necessary step to be undertaken in concert with nUV to assure safer environments for their occupants.

Interestingly, the results achieved from the air tests also showed a significant reduction of airborne contaminants (about 96%—Fig. 5).

It is appropriate to specify that this technology is not suitable for real-time treatment in the presence of people; the wavelength of 405 nm cannot make the air healthier in a short time, nor with the air dynamics, and because of airflows that can be generated by opening windows and doors. The usefulness of this approach should be understood in a context where, without air movement, in a “closed” context, it is desired to present occupants an environment in which, at the time of entry, the air is healthier.

LEDs having desired wavelengths allows for creating specific conditions of use; moreover, there is a progressive improvement of performance and reduction of expenditure that makes these systems more and more oriented to products addressed to large markets and no longer niche. However, it remains essential to know the limits of the technology and the context of its use so as not to brag about its benefits. In this regard, although nUV light at 405 nm is considered safe [29], LEDs can emit longer and shorter wavelengths than they are centered for, which could cause harmful effects, such as photoretinitis or pupil constriction [30, 31]. Wavelengths shorter than 400 nm are attentive to possible photobiological risk. It is important to know how to manage the problem of these exceeding doses through: i) the proper selection of LEDs with preference toward those that have a narrow wavelength around the peak and no or less radiant energy below the wavelength of 400 nm; ii) the adjustment of emission powers so as not to exceed the recommended doses over time so as not to engender photobiological hazards; iii) the management of exposure times relative to the distance of any operators from the light source so as not to have legislative barriers to their use [14]. Precautions should also be taken in the installation of light sources so that the irradiation of target surfaces is as homogeneous and disinfectionally effective as possible. To this end, it is conceivable to have more light sources for a better distribution of energy even if of lower radiant power compared to a few sources with a higher power. As shown by the study, going well beyond 5 J/cm^2^ does not produce significant increases in microbiological abatement. More light sources can also reduce the impact of the aforementioned shadow zone limitation, which is unavoidable in technologies that use light as a disinfectant source. In addition, using plastic caps to recreate darker areas near the sampling point allowed us to make a direct comparison, at the same light intensity, between the fully irradiated and shaded surfaces.

A detailed and clear risk management manual is another important element for the safe use of these light sources.

An inherent limitation of our research is the inability to know the type of bacterial/fungal species growing; however, a distinction was made between mesophile and psychrophile contamination, and the system was effective in both cases.

## Conclusion

The nUV ceiling lamps have proven to improve significantly environmental hygiene in a real setting such as a kindergarten. Appropriate precautions on installing and managing nUV light make this technological approach effective and safe, even in the presence of humans and pets.

## Data Availability

The data will be available upon reasonable request to corresponding author G.M.

## References

[1] Guidelines for non-pharmaceutical interventions to reduce the impact of COVID-19 in the EU/EEA and the UK. 24 September 2020. ECDC: Stockholm; 2020.

[2] Cauchemez S, Bhattarai A, Marchbanks TL, Fagan RP, Ostroff S, Ferguson NM, Swerdlow D, The Pennsylvania H1N1 working group, Sodha SV, Moll ME, et al. Role of social networks in shaping disease transmission during a community outbreak of 2009 H1N1 pandemic influenza. Proc Natl Acad Sci. 2011;108:2825–2830. 10.1073/pnas.1008895108. 10.1073/pnas.1008895108 PMC3041067 21282645

[3] Read JM, Zimmer S, Vukotich C, Schweizer ML, Galloway D, Lingle C, Yearwood G, Calderone P, Noble E, Quadelacy T (2021). Influenza and other respiratory viral infections associated with absence from school among schoolchildren in Pittsburgh, Pennsylvania, USA: a cohort study. BMC Infect Dis.

[4] Chao DL, Elizabeth Halloran M, Longini IM (2010). School opening dates predict pandemic influenza A(H1N1) outbreaks in the United States. J Infect Dis.

[5] COVID-19 in children and the role of school settings in transmission - Second update. Available online: https://www.ecdc.europa.eu/en/publications-data/children-and-school-settings-covid-19-transmission. Accessed on 18 Oct 2021.

[6] Doyle T, Kendrick K, Troelstrup T, Gumke M, Edwards J, Chapman S, Propper R, Rivkees SA, Blackmore C (2021). COVID-19 in primary and secondary school settings during the first semester of school reopening — Florida, August–December 2020. MMWR Morb Mortal Wkly Rep.

[7] Hershow RB, Wu K, Lewis NM, Milne AT, Currie D, Smith AR, Lloyd S, Orleans B, Young EL, Freeman B (2021). Low SARS-CoV-2 transmission in elementary schools — Salt Lake County, Utah, December 3, 2020–January 31, 2021. MMWR Morb Mortal Wkly Rep.

[8] Deshpande A, Mana TSC, Cadnum JL, Jencson AC, Sitzlar B, Fertelli D, Hurless K, Kundrapu S, Sunkesula VCK, Donskey CJ (2014). Evaluation of a sporicidal peracetic Acid/Hydrogen peroxide-based daily disinfectant cleaner. Infect Control Hosp Epidemiol.

[9] St. Denis TG, Dai T, Izikson L, Astrakas C, Anderson RR, Hamblin MR, Tegos GP (2011). All you need is light: antimicrobial photoinactivation as an evolving and emerging discovery strategy against infectious disease. Virulence.

[10] Messina G, Della Camera A, Ferraro P, Amodeo D, Corazza A, Nante N, Cevenini G (2021). An emerging innovative UV disinfection technology (part II): virucide activity on SARS-CoV-2. IJERPH.

[11] Casini B, Tuvo B, Cristina ML, Spagnolo AM, Totaro M, Baggiani A, Privitera GP (2019). Evaluation of an Ultraviolet C (UVC) light-emitting device for disinfection of high touch surfaces in hospital critical areas. IJERPH.

[12] Bosco R, Gambelli S, Urbano V, Cevenini G, Messina G. An integrated approach for better contamination control in hospital settings. Ig Sanita Pubbl . 2021; 77:404–413. 33883750

[13] Blatchley III E and Peel M. 2001. Disinfection by ultraviolet irradiation. In: Block, SS ed. Disinfection, sterilization, and preservation. Philadealphia: Lippincott Williams and Wilkins. p. 823- 829.

[14] Guidelines on limits of exposure to ultraviolet radiation of wavelengths between 180 nm and 400 Nm (Incoherent optical radiation): Health Phys. 2004;87:171–186. 10.1097/00004032-200408000-00006. 10.1097/00004032-200408000-00006 15257218

[15] Hamblin MR, Hasan T (2004). Photodynamic therapy: a new antimicrobial approach to infectious disease?. Photochem Photobiol Sci.

[16] Ochsner M (1997). Photophysical and photobiological processes in the photodynamic therapy of tumours. J Photochem Photobiol, B.

[17] Maclean M, Murdoch LE, MacGregor SJ, Anderson JG (2013). Sporicidal effects of high-intensity 405 Nm visible light on endospore-forming bacteria. Photochem Photobiol.

[18] Jankowska KI, Nagarkatti R, Acharyya N, Dahiya N, Stewart CF, Macpherson RW, Wilson MP, Anderson JG, MacGregor SJ, Maclean M (2020). Complete inactivation of blood borne pathogen Trypanosoma Cruzi in stored human platelet concentrates and plasma treated with 405 Nm violet-blue light. Front Med.

[19] McKenzie K, Maclean M, Timoshkin IV, Endarko E, MacGregor SJ, Anderson JG (2013). Photoinactivation of bacteria attached to glass and acrylic surfaces by 405 Nm light: potential application for biofilm decontamination. Photochem Photobiol.

[20] Wang Y, Ferrer-Espada R, Baglo Y, Gu Y, Dai T (2019). Antimicrobial blue light inactivation of *Neisseria Gonorrhoeae* : roles of wavelength, endogenous photosensitizer, oxygen, and reactive oxygen species. Lasers Surg Med.

[21] Maclean M, MacGregor SJ, Anderson JG, Woolsey G (2009). Inactivation of bacterial pathogens following exposure to light from a 405-nanometer light-emitting diode array. Appl Environ Microbiol.

[22] Bache SE, Maclean M, MacGregor SJ, Anderson JG, Gettinby G, Coia JE, Taggart I (2012). Clinical studies of the high-intensity narrow-spectrum light environmental decontamination system (HINS-Light EDS), for continuous disinfection in the burn unit inpatient and outpatient settings. Burns.

[23] Maclean M, MacGregor SJ, Anderson JG, Woolsey GA, Coia JE, Hamilton K, Taggart I, Watson SB, Thakker B, Gettinby G (2010). Environmental decontamination of a hospital isolation room using high-intensity narrow-spectrum light. J Hosp Infect.

[24] Murdoch LE, Maclean M, Endarko E, MacGregor SJ, Anderson JG (2012). Bactericidal effects of 405 Nm light exposure demonstrated by inactivation of *Escherichia, Salmonella, Shigella, Listeria, and Mycobacterium* species in liquid suspensions and on exposed surfaces. Sci World J.

[25] Andrady AL, Hamid SH, Hu X, Torikai A (1998). Effects of increased solar ultraviolet radiation on materials. J Photochem Photobiol, B.

[26] Wang Y, Ferrer-Espada R, Baglo Y, Gu Y, Dai T (2019). Antimicrobial blue light inactivation of Neisseria Gonorrhoeae: roles of wavelength, endogenous photosensitizer, oxygen, and reactive oxygen species. Lasers Surg Med.

[27] Leanse LG, Zeng X, Dai T (2021). Potentiated antimicrobial blue light killing of methicillin resistant Staphylococcus Aureus by Pyocyanin. J Photochem Photobiol B.

[28] Leanse LG, Dong P-T, Goh XS, Lu M, Cheng J-X, Hooper DC, Dai T (2020). Quinine enhances photo-inactivation of gram-negative bacteria. J Infect Dis.

[29] Liu X, Chang Q, Ferrer-Espada R, Leanse LG, Goh XS, Wang X, Gelfand JA, Dai T (2020). Photoinactivation of Moraxella Catarrhalis using 405-Nm blue light: implications for the treatment of otitis media. Photochem Photobiol.

[30] Matsumura Y, Ananthaswamy HN (2004). Toxic effects of ultraviolet radiation on the skin. Toxicol Appl Pharmacol.

[31] Foster RG (2009). Professor of circadian neuroscience, and head, nuffield laboratory of ophthalmology, university of oxford the ‘third’ photoreceptor system of the eye – photosensitive retinal ganglion cells. Eur Ophthalmic Review.

